# Descriptive epidemiology of hereditary musculoskeletal and limb defects in the isolated population of Chitral, North-West Pakistan

**DOI:** 10.12669/pjms.315.7594

**Published:** 2015

**Authors:** Saif Ullah, Javid Iqbal Dasti, Sajid Malik

**Affiliations:** 1 Saif Ullah, Human Genetics Program, Department of Animal Sciences, Faculty of Biological Sciences, Quaid-i-Azam University, 45320 Islamabad, Pakistan; 2 Javid Iqbal Dasti, Department of Microbiology, Faculty of Biological Sciences, Quaid-i-Azam University, 45320 Islamabad, Pakistan; 3 Sajid Malik, Human Genetics Program, Dept. of Animal Sciences, Faculty of Biological Sciences, Quaid-i-Azam University, 45320 Islamabad, Pakistan

**Keywords:** Musculoskeletal anomalies, Limb defects, Genetic epidemiology, Descriptive epidemiology, Chitral population, Pakistan

## Abstract

**Objectives:**

Musculoskeletal and limb defects (MLDs) are the major categories in hereditary anomalies and are a significant source of the disabilities. This study aimed at elucidating the nature and pattern of MLDs prevalent in Chitral district, which is an isolated population in the North-West of Pakistan.

**Methods:**

A cross-sectional epidemiological study was conducted in Chitral and subjects/families with MLDs were ascertained from public places, hospitals and door-to-door visits. The phenotypic manifestations, expressivity, sporadic/familial presentations, isolated/syndromic nature, inheritance pattern, and socio-demographic attributes, of MLDs were observed.

**Results:**

A total of 153 independent subjects/families with certain types of MLDs were recruited. The MLDs were classified into 9 major and 22 minor entities. In this cohort, polydactyly was observed to be overwhelmingly common (71%), followed by syndactyly and absence limb deformities. The majority of the cases (78%) had sporadic nature, 93% anomalies had isolated presentations; upper limbs were more commonly affected than the lower limbs; and unilateral cases were twice in ratio than bilateral. The majority of the malformations had milder phenotypes, however, 17% of the MLDs were severe in nature and resulted in certain types of disability, compromising the normal life of the subject.

**Conclusion:**

This research witnesses a distinctive pattern of MLDs in Chitral, which has not been reported for any other population of Pakistan so far. Further studies are required to observe the molecular etiologies of these malformations and to offer rapid diagnosis and genetic counseling.

## INTRODUCTION

The majority of the Mendelian or monogenic diseases are relatively rare individually, but collectively they become quite common. With the advent of better surveillance and stringent interventions infectious/communicable diseases are declining globally, however, hereditary and genetic disorders emerge as a major burden on the healthcare-systems.^1^ Musculoskeletal and limb defects (MLDs) for instance, are one of the prominent types of malformations among the congenital and hereditary anomalies, and are a major source of disability.^2^

MLDs may have mild to severe nature. The anomalies which are restricted to one organ-system and do not seriously affect the quality of life may be considered as milder types. These include for instance, digit defects like polydactyly, syndactyly, brachydactyly, etc. Severe anomalies like dwarfisms render long-term disabilities and are source of mortality in many instances.^2^,^3^ Population based studies are important to appreciate the burden of MLDs and to propose strategies for their management.

In Pakistan, high prevalence of genetic disorders has been reported. High rate of consanguinity, overlapping generations and stable communities have led to multigenerational pedigrees with several individuals exhibiting rare genetic disorders.^4^,^2^ Studies have shown that MLDs are one of the major categories of disorders among the total malformations. Due to inadequate diagnostic, management and rehabilitation facilities the burden of these disorders is greater in Pakistan than in Western countries. Better understanding of the types and nature of malformations is the pilot step towards the public health policy making. To this end, the present descriptive cross-sectional epidemiological study was carried out on MLDs in the population of the Chitral district, which is a remote and isolated population in the North-West of Pakistan.

## METHODS

This cross-sectional epidemiological study on MLDs was conducted during 2012-2013 in Chitral district of Khyber Pakhtunkhwa province. Subjects/families with certain types of MLDs were ascertained by visiting public places like educational institutes and community centers and through door-to-door surveys.

An informed consent was obtained, either from the individuals or their parents. For each individual, detailed data were obtained on demographic variables. Each subject underwent a clinical and physical examination with the help of a local medical practitioner. Detailed pedigrees were drawn from the familial cases. However, only the index subject in each family was included in the analyses. The study was approved by the Ethical Review Committee of the Quaid-I-Azam University, Islamabad.

MLDs were characterized with the help of a resident medical officer and by employing the standard medical databases.^5^,^6^ Digit defects like polydactyly and syndactyly were further characterized into well-described entities.^7^,^8^ Descriptive summaries were generated and the departure from random distributions was evaluated with χ^2^ and Fisher’s exact test statistics.

## RESULTS

### Spectrum of MLDs

**Fig.1:**
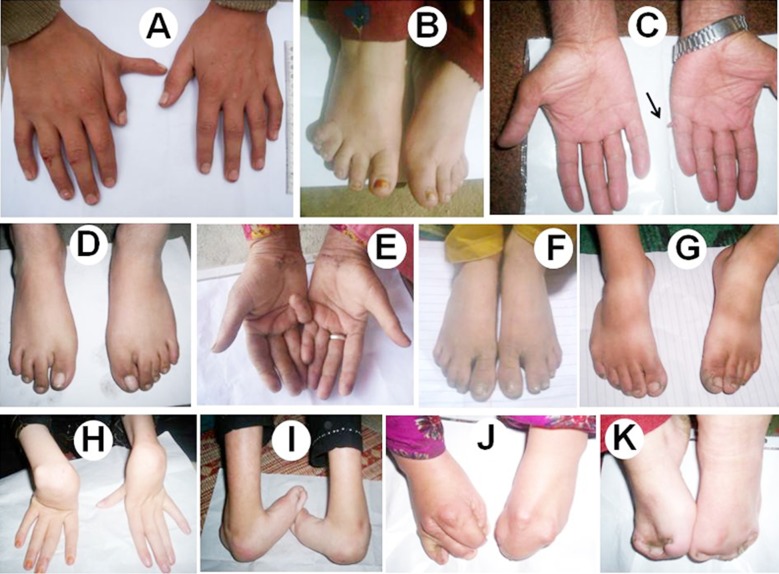
Phenotypic presentation of hereditary limb defects. A. Preaxial polydactyly of right hand. B. Postaxial polydacyly of right foot. C. Postaxial polydactyly (small knob like) of left hand. D. Oligodactyly in right foot. E. Unilateral camptodactyly of 5th finger in right hand. F. Bilateral partial cutaneous fusion of 2nd and 3rd toes. G. Talipes in right foot. H. Contracture deformity in both hands. I. Severe talipes in both feet. J-K. Extreme fusion of all digits.

There were 153 independent subjects/families ascertained with MLDs. These malformations were classified into nine broad categories, and among those polydactyly had the highest representation (n=109; 71%; proportion: 0.7124; 95%-CI: 0.6407-0.7841), followed by syndactyly (n=14; 9%; prop.: 0.0915; 95% CI: 0.0458-0.1372), absence deformities (n=9), musculoskeletal defects (n=6), talipes (n=5), contracture anomalies (n=4), brachydactyly (n=2), leg defects (disproportionate leg length; n=2), and overriding toe (n=2) (Table-I; Fig.1). The majority of the MLDs had a sporadic presentation (n=120; 78%), while familial cases were 22%. In polydactyly, postaxial type-B was the most common malformation (n=44), followed by preaxial type-I (n=43). There were five syndactyly types and syndactyly type-1a (zygodactyly) was the most prevalent type (n=9).

**Table-I:** Sub-types of the five major MLDs: distribution with respect to gender and familial/sporadic nature.

Malformation	Index subject		Familial attributes		Total No.	OMIM*
	Male	Female	Familial	Sporadic		
***Polydactyly***	76	33	22	87	109	
Preaxial type-I	29	14	5	38	43	174400
Preaxial type-III	1	0	1	0	1	174600
Preaxial type-IV	2	0	0	2	2	174700
Postaxial type-A	13	6	5	14	19	174200
Postaxial type-B	31	13	11	33	44	174200
***Syndactyly***	9	5	3	11	14	
Type-1a	5	4	3	6	9	609815
Type-1b	2	0	0	2	2	185900
Type-1c	1	0	0	1	1	
Type-II	1	0	0	1	1	186000
Type-VII	0	1	0	1	1	212780
***Absence deformities***	7	2	2	7	9	
Amputations (transverse/longitudinal)	4	1	1	4	5	217100
Oligodactyly	2	0	0	2	2	
Symbrachydactyly	1	0	1	0	1	
Constriction rings/symbrachydactyly	0	1	0	1	1	
***Musculoskeletal anomalies***	2	4	2	4	6	
Dwarfism	1	1	0	2	2	100800
Neuromuscular/muscular atrophy	1	3	2	2	4	310200
***Talipes***	4	1	1	4	5	119800
Talipes calcaneovarus	2	0	0	2	2	
Talipes varus	1	1	1	1	2	
Talipes valgus	1	0	0	1	1	
***Contracture anomalies***	0	4	0	4	4	
***Brachydactyly***	1	1	1	1	2	
***Leg defects***	2	0	0	2	2	
***Overriding toe***	2	0	2	0	2	
**Total (in major categories)**	103	50	33	120	153	

* most closest definitions found in the OMIM database.

### Affected male-to-female ratio

Male subjects were found to be commonly affected than the females in most of the ascertainment categories. For instance, in the total index cases the frequency of affected males was twice than that of females (M:F= 2.1:1). Among the sporadic cases (n=120), 84 males and 36 females were observed (2.3:1). In the familial index cases (n=33), there were 19 males and 14 females.

### Expressivity analysis: Involvement of upper and lower limbs

**Fig.2:**
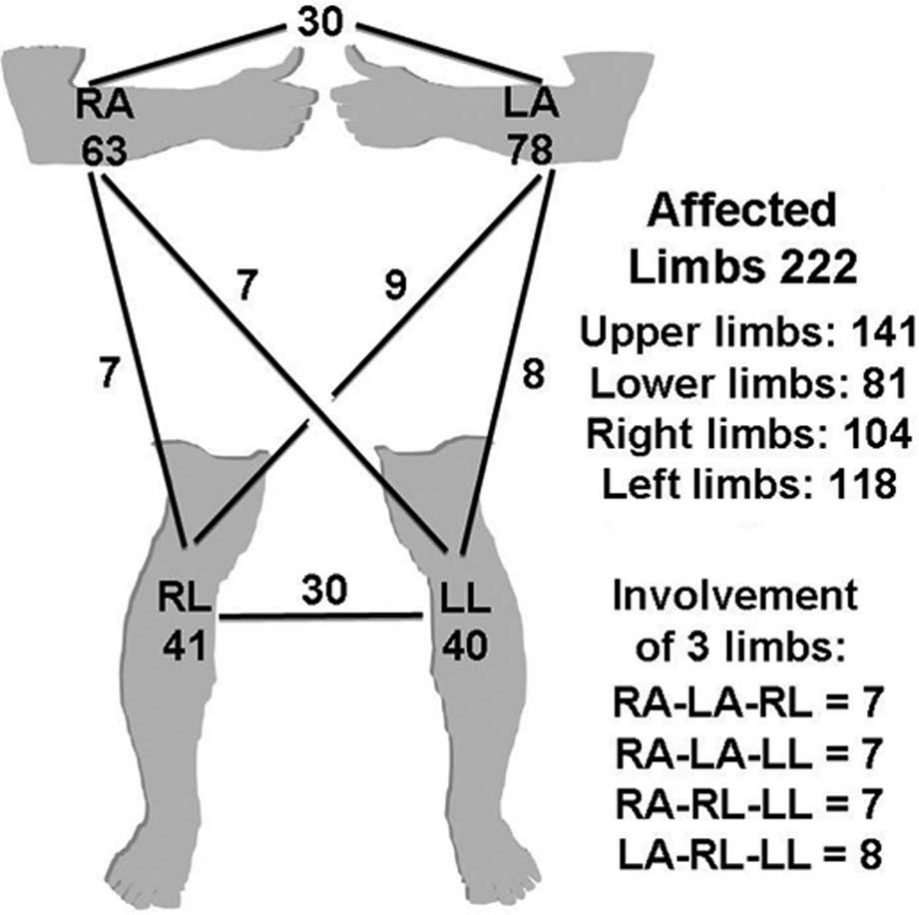
Line drawing depicting the frequency of involvement of each limb and the combinations of two affected limbs. (RA=right arm; LA=left arm; RL=right leg; LL=left leg)

The 153 index subjects had a total of 222 affected limbs (Table-II). The upper limbs were more commonly affected (n=141) than the lower limbs (n=81) (p=0.004) (Fig.2). In the upper limbs, left arm was slightly more affected than the right (78 vs. 63, respectively). In the lower limbs, right and left legs were almost equally involved. Furthermore, only the upper limbs were affected in 102 cases, only the lower limbs in 42 subjects, and both upper and lower limbs in 9 instances. Additionally, only one limb was affected in 99 cases, any two limbs in 46, any three limbs in one subject, and all four limbs were observed to be affected in seven cases.

**Table-II:** Pattern of affected limbs and the combination of involved limbs in subjects with MLDs.

Malformation	No. of cases (n=153)	Total affected limbs (n=222)	Upper limb (n=141)		Lower limb (n=81)		No. of cases with involvement of ….			No. of limbs involved (in 153 cases)			
			RA	LA	RL	LL	Arms only	Legs only	Both	Any 1	Any 2	Any 3	All 4
Polydactyly	109	136	48	60	14	14	88	21	0	82	27	0	0
Syndactyly	14	30	3	4	12	11	2	9	3	3	8	1	2
Absence deformities	9	12	4	6	2	0	7	1	1	6	3	0	0
Musculoskeletal defects	6	22	5	5	6	6	0	1	5	0	1	0	5
Talipes	5	8	0	0	4	4	0	5	0	2	3	0	0
Contracture anomalies	4	4	2	2	0	0	4	0	0	4	0	0	0
Brachydactyly	2	4	1	1	1	1	1	1	0	0	2	0	0
Leg defects	2	3	0	0	1	2	0	2	0	1	1	0	0
Overriding toe	2	3	0	0	1	2	0	2	0	1	1	0	0
Total	153	222	63	78	41	40	102	42	9	99	46	1	7

RA=right arm, LA=left arm, RL=right leg, LL=left leg.

### Familial and sporadic cases of MLDs

In 33 familial cases, the malformation was witnessed to segregate in one (n=14) or two generations (n=15). Across all pedigrees, the malformations were segregating 1.70±0.68 generations on average. Furthermore, the mean affected sibship per pedigree was calculated to be 1.70±0.68, while affected subjects per family averaged 3.06±1.77 (data not shown).

The 120 sporadic cases were analyzed with respect to the parity/gravida of the index subject and the size of his/her normal sibship. The majority of index subjects belonged to first parity (43%; n=51), followed by 2^nd^ (37%; n=44), and 3^rd^ gravida (13%; n=15). The parity in all sporadic cases averaged 1.91±1.05.

### Associated malformations in the index subjects and their families

MLDs had essentially isolated presentations in 93% (n=142) cases. Eleven index subjects were observed to have 16 associated malformations, the most common of which were syndactyly (n=5) and camptodactyly (n=3). There were 13 occasions in which certain other malformation was witnessed in the family members of the index subject.

### Demographic distribution of subjects/families

Distribution of subjects/families was established along the socio-demographic variables of the Chitrali population (Table-III). In the majority of these variables, the distributions were statistically not significant in the male/female and sporadic/familial samples. However, statistically significant differences were observed in the distributions in variables like literacy and family type.

**Table-III:** Demographic distribution of 165 index subjects with MLDs (with respect to gender and familial/sporadic nature).

Demographic variable	Index subject		Familial attributes		Total
	Male	Female	Familial	Sporadic
*Origin*					
Rural	101	50	33	118	151
Urban	2	0	0	2	2
Total	103	50	33	120	153
*Age range (yrs)*					
≤19	73	25	16	82	98
20-39	15	10	8	17	25
≥40	15	15	9	21	30
χ^2^=	p=0.0304*			p=0.1075	
*Caste / Ethnicity*					
Zondre	23	7	9	21	30
Rizeae	16	3	7	12	19
Paane	10	5	2	13	15
Khoshwahte	10	4	1	13	14
Dashmane	8	5	3	10	13
Waliye	9	4	2	11	13
Others (<13)	27	22	9	40	49
χ^2^=	p=0.2932			p=0.3385	
*Literacy level (age ≥6 yrs; n=126)*					
Illiterate	11	18	12	17	29
Literate	71	26	19	78	97
χ^2^=	p=0.0005*			p=0.0168*	
*Family/house-hold type*					
Nuclear	16	13	1	28	29
Extended	87	37	32	92	124
χ^2^=	p=0.1213			p=0.0084*	

*distribution was statistically significant. Categories with non-zero values were used in the analyses.

## DISCUSSION

In the present cohort, majority of the MLDs had rather milder nature. However, 17% (n=26) malformations had severe nature. These included absence deformities, musculoskeletal defects, talipes, contracture anomalies and leg defects. It was observed that these anomalies resulted in certain types of restrictions in the normal life functions of the subjects and none of them had undergone cosmetic improvements. Nonetheless, in all the ascertainment categories of recruited subjects (i.e., index subjects, familial/sporadic, total affected family members), a high male-to-female ratio was observed. A strong sex distortion has been reported in other congenital and hereditary malformations. For instance, in case of talipes, males are twice in proportion as compared to females.^6^ In polydactyly, males are more commonly affected then the females.^9^

There were nine major classes of MLDs in the present sample which could be further categorized into 20 sub-types. In an epidemiological study in Southern Pakistan, nine major types of MLDs were witnessed, and polydactyly and syndactyly were the most common malformations.^10^ Polydactyly was also the most prevalent type in the present study. Polydactyly cases were further characterized into five well-characterized preaxial and postaxial entities. Postaxial type-B (OMIM-174200) was observed to be the most prevalent type, followed by preaxial type-I (OMIM-174400). Collectively, postaxial types were more common. However, postaxial types were common in the South American cohort.^11^ Similar to an epidemiological study carried out by Castilla *et al.^9^* in South America, we also observed a preponderance of affected males, low frequency of familial occurrence, involvement of upper limbs more frequently than the lower limbs, and left arm frequently more affected than the right. Furthermore, there were five distinct types of syndactylies in the present study and zygodactyly (OMIM-609815) was observed to be the most common type. Zygodactyly has been observed to be the most common type in several independent epidemiological studies with a prevalence of 2-3/10,000.^5^

One of the interesting findings of the present study is the highest representation of digit anomalies among the MLDs. Polydactyly, which was overwhelmingly common in this dataset, had mainly sporadic presentations. In an extended cohort of 313 subjects with polydactyly from Pakistan, 65% cases were observed to be sporadic.^4^ The majority of isolated congenital digital anomalies occur spontaneously without any family history. This has led researchers to search for environmental etiologic factors. By employing a very large dataset, Man and Chang^12^ demonstrated that maternal smoking during pregnancy was a statistically significant risk factor for having a newborn with polydactyly, syndactyly or adactyly.

Chitralis are traditionally conservative and it was difficult to interact with the community without a local resource person familiar with Khower language and their customs. Moreover, the climate is extreme for several months of the year, especially the harsh winter do not allow much economic activity in the area. Because of cultural and religious influences women’s mobility is restricted. Hence, a general epidemiological study focusing all types of hereditary malformations was not possible (this may also partly explain the high representation of male subjects among the index cases).

## CONCLUSION

This study is the first description of MLDs in the remote and isolated population of Chitral. One of the major advantages of the present study is a sufficiently large and random sample ascertained from 24 different sites. The current study, however, does not provide the true prevalence and incidence rates of limb anomalies which could be useful in estimating the frequencies of mutation(s) and heterozygote(s) in the populations. Moreover, this study highlights a significant proportion of MLDs cases which have sporadic and isolated nature, and warrants further case-control studies in order to dissect the potential genetic and environmental causes.
